# Correlation between Nuclear Morphology and Adipogenic Differentiation: Application of a Combined Experimental and Computational Modeling Approach

**DOI:** 10.1038/s41598-019-52926-8

**Published:** 2019-11-08

**Authors:** Andrew McColloch, Manoochehr Rabiei, Parisa Rabbani, Alan Bowling, Michael Cho

**Affiliations:** 1 University of Texas at Arlington, Department of Biomedical Engineering, Arlington, 76010 USA; 2 University of Texas at Arlington, Department of Mechanical and Aerospace Engineering, Arlington, TX 76010 USA

**Keywords:** Biological fluorescence, Biomedical engineering

## Abstract

Stem cells undergo drastic morphological alterations during differentiation. While extensive studies have been performed to examine the cytoskeletal remodeling, there is a growing interest to determine the morphological, structural and functional changes of the nucleus. The current study is therefore aimed at quantifying the extent of remodeling of the nuclear morphology of human mesenchymal stem cells during biochemically-induced adipogenic differentiation. Results show the size of nuclei decreased exponentially over time as the lipid accumulation is up-regulated. Increases in the lipid accumulation appear to lag the nuclear reorganization, suggesting the nuclear deformation is a prerequisite to adipocyte maturation. Furthermore, the lamin A/C expression was increased and redistributed to the nuclear periphery along with a subsequent increase in the nuclear aspect ratio. To further assess the role of the nucleus, a nuclear morphology with a high aspect ratio was achieved using microcontact-printed substrate. The cells with an elongated nuclear shape did not efficiently undergo adipogenesis, suggesting the cellular and nuclear processes associated with stem cell differentiation at the early stage of adipogenesis cause a change in the nuclear morphology and cannot be abrogated by the morphological cues. In addition, a novel computational biomechanical model was generated to simulate the nuclear shape change during differentiation and predict the forces acting upon the nucleus. This effort led to the development of computational scaling approach to simulate the experimentally observed adipogenic differentiation processes over 15 days in less than 1.5 hours.

## Introduction

Adult mesenchymal stem cells (MSCs) are of great interest in tissue engineering and in clinical applications due to their capability to differentiate into multiple lineages including bone, adipose, and cartilage^1^. This differentiation potential is unique in stem cells and highly sought after as a method of regenerating the damaged or lost tissue. Typically, MSC differentiation is induced by the lineage-specific chemical stimuli, with the ratio of growth factors optimized for the desired tissue^2^. In addition, multiple laboratories have investigated the influence of cellular morphology and substrate mechanical properties on MSC differentiation^3–6^.

While important strides have been made in elucidating the influence of the cytoskeletal mechanical properties on MSC differentiation, few have examined the effects of the nucleus. The nucleus is the stiffest organelle in the cell and is also responsible for cell mechanics^7^. Additionally, the nucleus houses the cell’s genetic code in the form of mobile chromatin structures, which shift as cell morphology changes. For example, it has been previously shown that DNA is wound into chromatin territories, with specific areas corresponding to specific genes^8–10^. The localization of genes within the nucleus is not random and have been demonstrated to be related to gene activation^11,12^, suggesting the reorganization of the nucleus is a prerequisite to initiate the reprogramming to a different cell lineage^13^.

Nuclear mechanical characteristics can be classified as viscoelastic, with two main contributors: DNA wound into chromatin, and the nuclear envelope proteins lamin A and C (LMNA)^14^. LMNA is the major structural protein in the nuclear envelope and is thought to be responsible for nuclear shape and chromosome positioning^15^. Defects in the LMNA gene have been associated with various human diseases known as laminopathies with several symptoms including progeria, muscular dystrophy, and cardiomyopathy^16^. Several laminopathies primarily affect mesenchymal tissues, including mesenchymal stromal cells, and can result in enhanced cellular senescence and differentiation potential^17,18^.

Several strides have been made in revealing the effect of nuclear shape change and lamin reorganization during cellular differentiation, including the influence of lamin A/C. For example, Verstraeten *et al*. demonstrated the changes in the nuclear lamin network during adipogenesis of mouse 3T3-L1 pre-adipocytes^19^. LMNA was shown to move from internuclear structures to the nuclear rim as adipogenesis continued. Interestingly, it was discovered that the fraction of cells expressing lamin increased during differentiation, a phenomenon also seen in human embryonic stem cells where B-type lamins are dominant in an undifferentiated state with eventual lamin A/C activation occurring during differentiation^20^. Furthermore, a point mutation in LMNA resulting in a common laminopathy disorder (p. R482W) has been shown to inhibit adipogenesis by de-regulating anti-adipogenic gene enhancers through an epigenetic mechanism^21^. LMNA has additionally been shown to be influenced by the mechanical properties of the substrate, with soft matrices reducing overall lamin levels^22^. Together, these bring a focus to the role of LMNA in adipogenic differentiation and its influence on controlling the underlying chromatin structure.

Potentially, repositioning of the chromatin structure necessary for lineage specificity of stem cells occurs via LMNA reorganization during stem cell differentiation, resulting in specific gene activation for lineage-directed differentiation. It is reasonable to postulate that DNA could be morphologically manipulated for such genetic activation. This morphology change, therefore, would be driven by LMNA and result in nuclear shape change. In this paper we monitored and quantified several morphological features of human MSC nuclei over time along with the expression of LMNA and correlated them with adipogenic differentiation markers. We also constructed a 2D numerical simulation model to elucidate the mechanical coupling of F-actins to the nucleus that appears to regulate changes in the shape and size of the nucleus. The high speed simulation was made possible through a multiscale approach that significantly reduced the computational cost (<1.5 hrs) while probing the nuclear membrane properties such as its stiffness. Experimental measurements of such nuclear biomechanical properties with certainty remain yet to be established.

## Results

**Figure 1:**
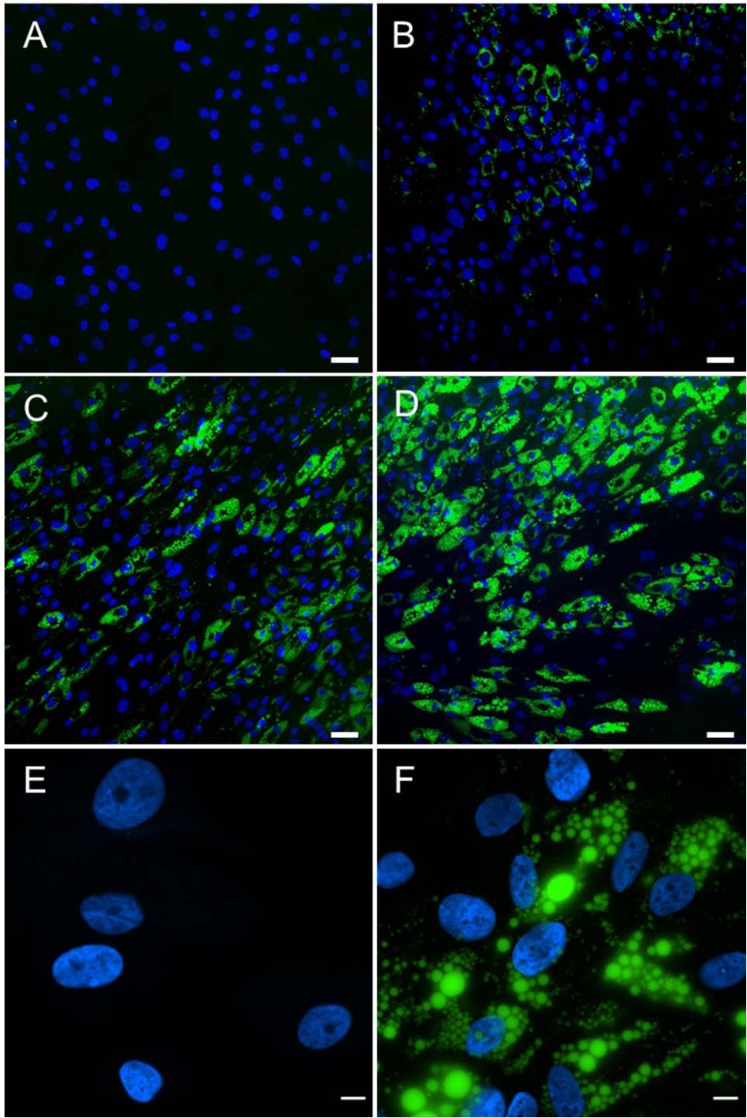
Adipogenic differentiation over 15 days. MSCs underwent differentiation toward adipocytes in AD medium. Fluorescent markers observed are DAPI (blue) and LipidTOX (green). Images shown depict days 1 (**A**), 5 (**B**), 9 (**C**), and 15 (**D**). Scale bar = 50 µm (**A** through **D**). In addition, high magnification (90×) images of the nuclei and lipid droplets in hMSC during adipogenic differentiation acquired at 1 (**E**) and 7 (**F**) days. Scale bar = 10 µm (**E**,**F**).

Human MSCs were seeded onto glass coverslips and induced to differentiate to adipocytes using previously described factors contained in the Adipogenic Differentiation (AD) medium. The extent of adipogenic differentiation was monitored and imaged using LipidTOX staining to visualize the accumulated lipids. Stem cells undergoing adipogenic differentiation at day 1 showed essentially no detectable accumulation of lipids (Fig. 1A). Lipid accumulation became increasingly visible at day 5 (Fig. 1B) and at day 9 (Fig. 1C). By day 15, the stem cells incubated with the AD medium demonstrated a high level of LipidTOX staining (Fig. 1D). The nuclei were also stained and imaged using DAPI. It is interesting to note that there still is a fraction of cells that showed no lipid accumulation even at day 15. To better visualize adipogenesis, images were acquired using a higher magnification microscope objective (90×) at day 1 (Fig. 1E) and day 7 (Fig. 1F). A few interesting observations should be noted. First, the lipid droplets were shown to be non-uniform. Second, the varying size and shape of nuclei were visible at day 7. There appears to be a correlation between the nuclear morphology and the level of lipid accumulation.

**Figure 2:**
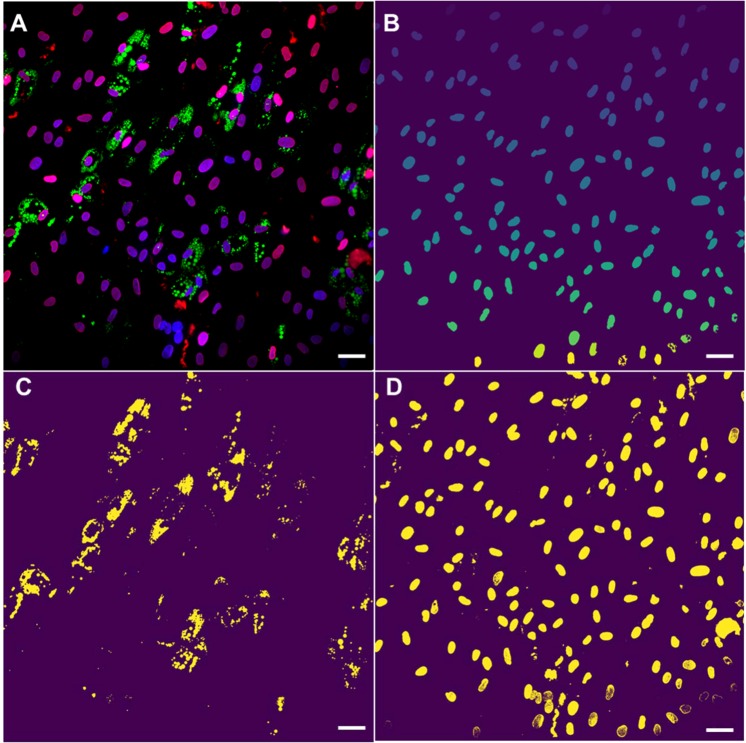
Python-generated images depicting masks for computer-assisted analysis of morphological and fluorescent data. (**A**) Original composite 3-color fluorescent microscope image- nuclei (blue), lipid (green), and LMNA (red). (**B**) Nuclei segmented and the size and shape determined after K-means clustering. Lipid droplets (**C)** and LNMA expression (**D**) segmented and separated from the original image. Images were taken from the same location after 9 days of differentiation using a 20X microscope objective. Rapid segmentation was achieved by the code to process thousands of cells without sacrificing accuracy. Scale bars = 50 µm.

To further examine and determine such a correlation between the nuclear morphology and the extent of adipogenesis, we developed a custom-built code to rapidly and accurately analyze a large number of stem cells undergoing adipogensis. Following image acquisition, fluorescent images were processed using code written in Python for the nuclei morphology, lipid production, and lamin A/C (LMNA) intensity. To increase statistical significance, a 20x microscope objective was used to capture between 75 and 100 cells in a single field of view. For example, one set of images taken at day 9 was further processed and analyzed (Fig. 2). Nuclei were segmented via the unsupervised learning algorithm K-means clustering, a method commonly utilized for cell segmentation^23^. Three-color fluorescent images were first merged (Fig. 2A), and signal from DAPI staining and background were separated, clustered nuclei were delineated, and finally the nuclear morphological characteristics were ascertained (Fig. 2B). Additionally, lipid accumulation via LipidTOX fluorescence and LMNA expression were also separated and analyzed by utilizing masks of the different fluorescent channels (Fig. 2C,D, respectively).

**Figure 3:**
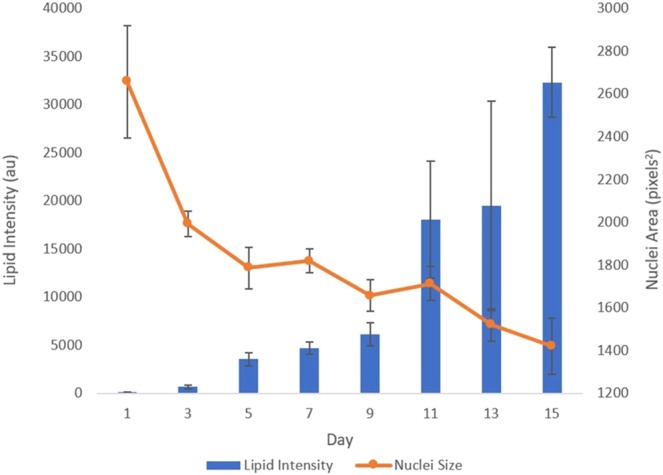
Correlation between the nucleus size and lipid deposition. Nuclei area, as assessed by the DAPI fluorescence, decreased over time, and the lipid production increased exponentially. A reduction in the nucleus size was notably evident by day 5, while lipid increased most significantly after day 9. Data presented as mean ± SEM from 10 independent experiments averaging over 600 cells per each experiment.

The images processed by the custom-built code allowed for rapid but more accurate quantification of the nuclear morphological parameters such as the nuclear size, aspect ratio and roundness. A significant decrease (~70%) in the nucleus size (*pixels*^2^) was observed within the first 5 days of adipogenesis (Fig. 3). The subsequent and further reduction in the nucleus size was not as pronounced in the next 10 days of adipogenesis. The accumulation of lipid droplets was monitored at the same time and fluorescently measured to assess the extent of adipogenesis. The accumulation of lipid droplets over 15 days appeared to have increased exponentially. However, unlike the changes we observed in the nuclear size, the lipid production was substantially increased after 9 days of adipogenic differentiation and appeared to lag behind the remodeling of nuclei. More than 80% of lipid deposition occurred following the most significant decrease in the nuclear area at day 5. The implication here may be that the nuclear remodeling and morphological modulation precede adipogenesis and therefore could be a prerequisite for the intended differentiation.

**Figure 4:**
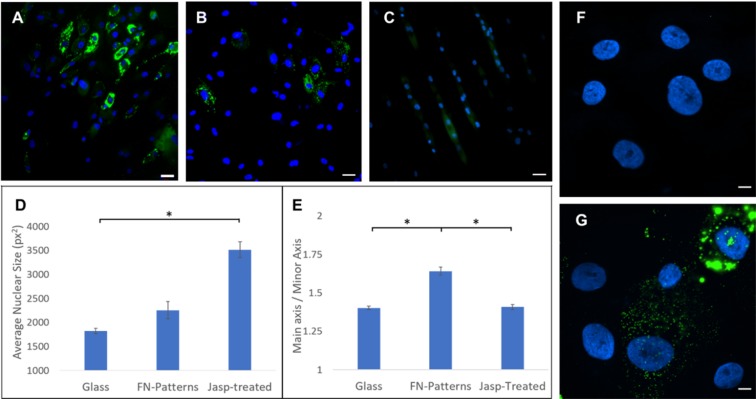
Comparison of adipogenic differentiation of hMSCs plated on glass (**A**, control), jasplakinolide-treated (**B**) and seeded on micro-contact printed surface. **(C**) Cells treated with the actin stablizing agent demonstrated significantly larger nuclear area vs. control. (**D**) Cells seeded on the patterned substrates showed similar nuclear area, but with an increased aspect ratio. (**E**) Nuclei in blue color and lipid expression in green color. Data represent mean ± SEM of 3 independent experiments. Scale bars = 50 µm. In addition, (**F**,**G**) show high magnification (90×) images of treated cells with jasplakinolide at day 1 and 7 of differentiation, respectively. In the jasplakinolide-treated cells, the size of nuclei was larger with a much diminished lipid accumulation. Scale bars = 10 µm. *Denotes p < 0.05.

Since changes in the nuclei are likely mediated by reorganization of the cytoskeleton^24–26^, two different methods were applied to influence the cytoskeleton and thereby alter the size and shape of the nucleus. First, the cells were treated with a pharmacological agent (jasplakinolde) to stabilize the actin structure that presumably resisted changes in the nucleus. Second, the cells were seeded onto a fibronectin-coated and microcontact-printed substrate to suppress adipogenesis. Fluorescent images at day 7 of adipogenesis show that both the jasplakinolde treatment and forced elongated cell seeding led to suppression or delay of the intended differentiation (Fig. 4A–C). Quantitative analysis of the nuclear morphology following the two treatments revealed that adipogenesis can be hindered by either keeping the nuclear size artificially large (Fig. 4D; jasplakinolde treatment) or by increasing the nuclear aspect ratio (Fig. 4E; elongated cell seeding). To better visualize the effect of actin cytoskeleton, images of lipid droplets and nuclei were acquired using a 90x magnification objective following the jasplakinolde treatment (Fig. 4F at day 1 and Fig. 4G at day 7). The results are consistent that the pharmacologically stabilized actin cytoskeleton hindered the intended differentiation. Moreover, to address potential impact of altered cell proliferation among treated and control groups, an MTT assay was performed. After seven days of differentiation, no significant differences were observed between the control and treated groups (data available but not shown).

**Figure 5:**
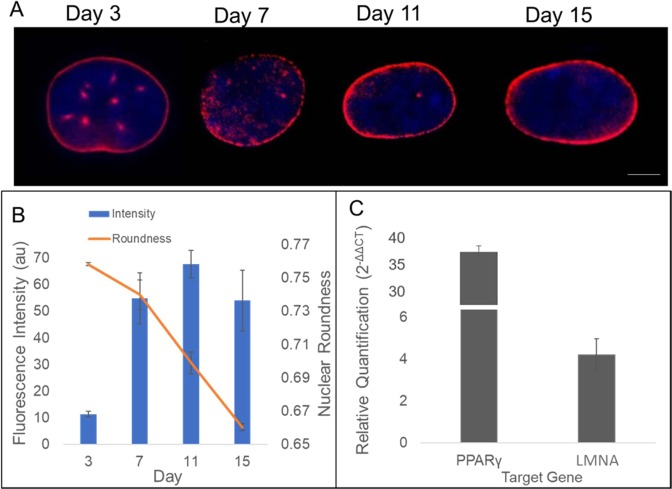
LMNA expression and distribution. **(A**) Fluorescent images of LMNA at different days of adipogenic differentiation. Images recorded using a 60X magnification objective. Scale bar = 5 µm. (**B**) The LMNA expression increases early during differentiation, while nuclear morphology (roundness) is steadily reduced. (**C**) qPCR data show relative fold changes of adipocyte maturation gene PPARγ and nuclear envelope gene LMNA. Data represent mean ± SEM of 3 independent experiments.

To further probe the influence of nuclear morphology on adipogenic differentiation, we monitored and quantified the intensity and distribution of a major nuclear envelope protein, LMNA. Using fluorescently conjugated antibody, the LMNA expression was shown to increase during differentiation as the nuclear remodeling occurred (Fig. 5A). The LMNA was observed to redistribute over time and became more prevalent along the periphery of the nuclei along with a reduction in punctate structures of the nuclear interior. As another measure of the nuclear morphology, the roundness was monitored and measured. The nuclear roundness, which is inverse of the aspect ratio, was linearly decreased over the 15 days of differentiation (Fig. 5B). Conversely, the LMNA fluorescence intensity sharply increased from day 3 to day 7 and then remained relatively unchanged. To probe the gene expression of LMNA during adipogenesis, total RNA was extracted at day 5 of differentiation, which corresponds to the day of the most significant decrease in nuclear area (see Fig. 3). The adipose gene peroxisome proliferator-activated receptor gamma (PPARγ) was used as a positive control due to its recognized upregulation (~35-fold) during adipogenesis^27^. Notably, LMNA mRNA production increased more than 4-fold compared to control (Fig. 5C). Taken together, the LMNA is up-regulated and also spatially redistributed to the nuclear membrane over time.

**Figure 6:**
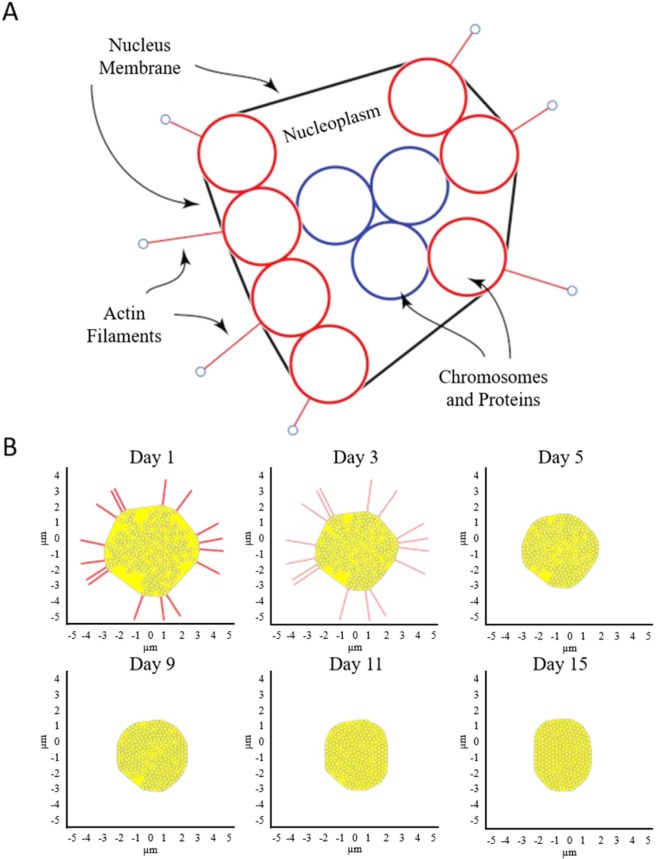
(**A**) Schematic of nucleus with actin filaments, membrane, cytoplasm, and nuclear components. (**B**) Frame stills from modeling simulation of nuclear shape during differentiation. The initial number of actin springs represented by the red lines is assumed to be 16. The cytoskeletal tension is released during maturation, allowing elastic LMNA to conform to a more compact shape. Interior beads represent repositioning of the nuclear components while extended red lines represent actin filaments dissociating from the nucleus over time. Axes indicate nuclear dimension in *µ*m.

**Figure 7:**
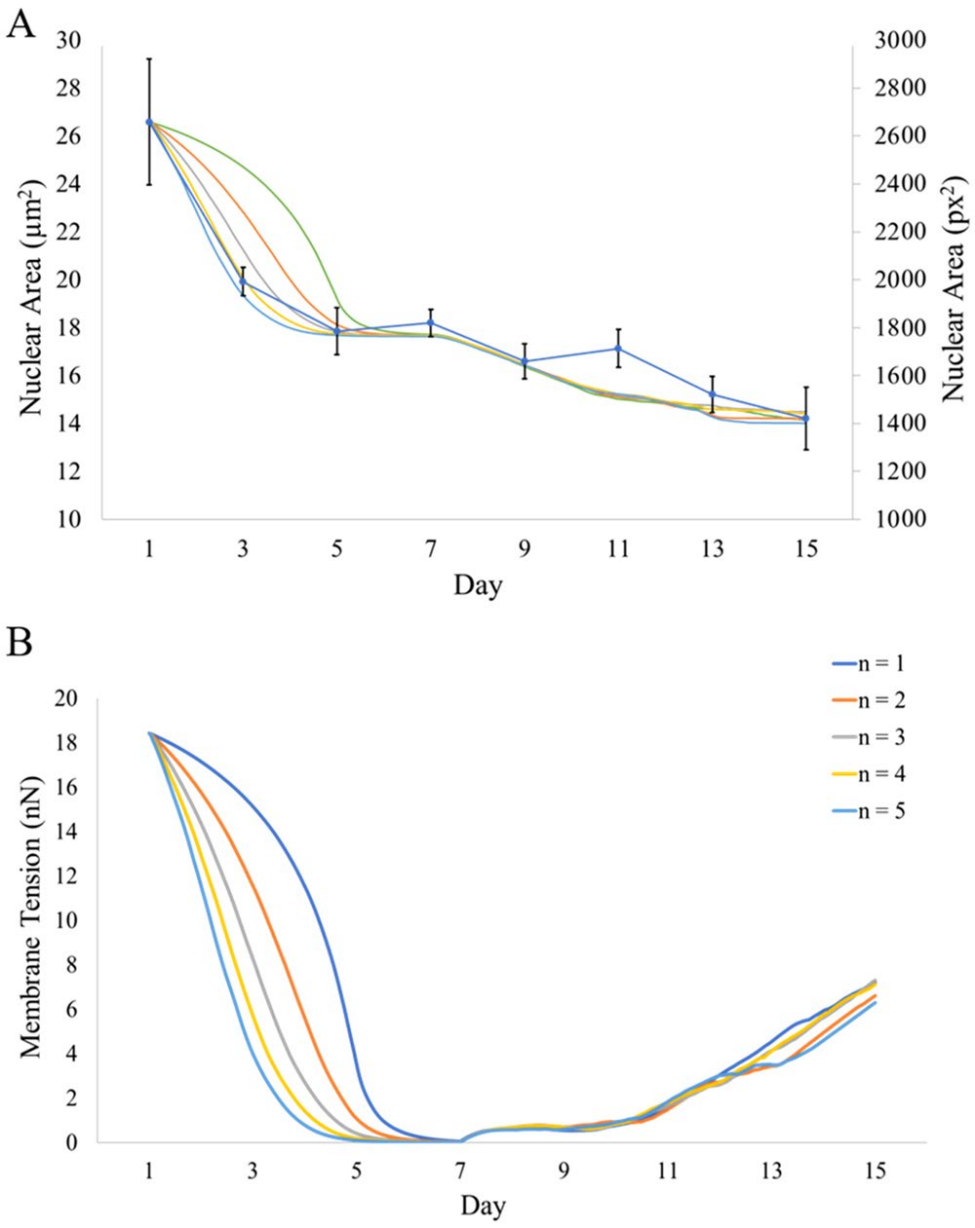
(**A**) Model-generated results of nuclear size demonstrating consistency with experimental data (blue curve with error bars) over 5 separate simulations. (**B**) Mathematical calculation of changes in the nuclear membrane tension (nN) as actin filaments reorganize and disappear. Results from 5 separate simulations are shown. Simulations are a result of increasing *n* from Eq. 1. It was found that the time dependence of 4^th^ power (*n* = 4 shown in yellow) most closely followed experimental data.

Numerical simulation was next utilized to better understand the dynamics of nuclear remodeling. Because experimental measurements of the nuclear biomechanics with certainty are lacking during adipogensis, the results from numerical simulation can provide important and quantitative mechanical environment in the nucleus. For simplicity and to significantly reduce computational time, the nucleus is assumed to be filled with beads that represent the nuclear components such as chromosomes and other structures. To model the cytoskeletal forces and their change, the actin filaments are attached to the nuclear membrane and allowed to dissociate from it over time (Fig. 6A). It is reasonable to assume that actin stress fibers keep the nucleus membrane under tension^28–30^. As differentiation proceeds and actins are rearranged, this tension is released, and therefore the nucleus undergoes morphological changes. After actin is depolymerized, the lipid droplets are formed that exert additional force on the nucleus. Several frame stills of the simulation were captured and shown to depict the actin tension over time as the actin tension was reduced while the lipid droplets accumulated (Fig. 6B). To validate the model, at least 5 simulations were carried out and the results were compared to the experimental data (Fig. 7A). The simulation model was able to faithfully reproduce the experimental data, demonstrating a good agreement. Using the model, the forces acting on the nucleus as a function of time may be simulated and predicted (Fig. 7B). For example, the initial nuclear membrane tension is found to be ~20 nN and decreases rapidly to zero as the actin cytoskeleton is reorganized during the first 7 days of differentiation. The membrane tension is then observed to rise to ~8 nN, because of the interaction between internal and external forces acting on the nuclear membrane. The external forces are caused by lipid accumulation, which squeeze the nucleus causing contraction. The internal forces are caused by resistance to compaction by the protein and chromosome structures within the nucleus.

## Discussion

As stem cells progress to fully differentiated lineages, they undergo several morphological changes. These changes occur both in the cytoskeleton as well as the nucleus. Several previous researchers have examined the actin remodeling that takes place during adipogenesis. Titushkin *et al*., described the rearranging of actin filaments to the cell perimeter and eventual depolymerization as maturation continued^31^. Others include the influence of substrate stiffness and its response to actin organization on differentiation potential. Indeed, a modified substrate enhances the differentiation efficiency of MSCs to specific cell types^3–5^. However, studies on remodeling of the nucleus are scarce.

The nucleus contributes to the overall mechanical properties of the cell by being one of the largest and stiffest organelles^7^. The nucleus is also the repository for the cell’s genetic code, which must be manipulated for gene activation. As demonstrated in Fig. 3, the nucleus decreases in size over time during differentiation to mature adipocytes. Interestingly, the nuclear size reduction occurs mostly by day 5, with 80% of reduction occurring by this time point. The nuclear shrinkage precedes the exponential growth of fat deposition – a marker of mature adipocytes – suggesting the nuclear reorganization must occur prior to activation of the intricate machinery that promotes mature adipocytes. This is consistent with the findings that hMSC adipogenic differentiation occurs in two distinct parts; (1) lineage direction into pre-adipocytes and (2) adipocyte maturation^32–35^. Moreover, along with a reduction in nuclear size, we also observe a change in overall morphology of nucleus as adipogenesis continues. Differentiation brings about an increase in the aspect ratio (e.g., reciprocal decrease in roundness) of the nucleus. We compared the timing of the nuclear shape change with that of the expression of the nuclear structural protein lamin A/C and found an inverse relationship. The LMNA protein expression appeared to increase as the nuclear morphology was altered to an increased aspect ratio (Fig. 5). Additionally, RT-qPCR experiments demonstrated a larger than 4-fold increase in production of LMNA mRNA when compared to control. LMNA exists as a network of intermediate filaments to provide structure and support for the nuclear envelope^36^. As a major driver of nuclear configuration, it stands to reason that an upregulation of LMNA may actively manipulate the nuclear shape. Indeed, LMNA structures have been shown to directly control chromosome positioning^37^, which in turn result in gene activation^38^. Fluorescent images of LMNA suggest a redistribution, as well as upregulation, of LMNA as adipogenesis continues. LMNA reorients along the nuclear periphery accompanied by a reduction in internuclear punctuates. This redistribution follows in line with published results which demonstrated a reorganization of the lamin network from internuclear structures during early differentiation to localization along the nuclear rim^19^. However, similar groups investigating nuclear structural rearrangement show an inverse phenomenon in expression levels of LMNA. Here, we demonstrate LMNA levels redistribute and increase in expression, while several researchers describe an overexpression of lamin as inhibiting adipocyte differentiation and lipid synthesis^20,21^. Verstraeten *et al*., also described an overall loss of lamin proteins after 18 days of adipocyte differentiation when compared to non-differentiated preadipocytes^19^. One potential explanation to this observation is the length of differentiation performed in regards to cell type. Several examples explained above made use of the commonly studied mouse 3T3-L1 preadipocyte cell line, which can attain a fully differentiated state in as little as 10 days^39^. Human mesenchymal stem cells derived from bone marrow are however not primed for adipocyte differentiation and may therefore require longer differentiation times to observe the full effect. Indeed, we did observe a change in expression and localization of LMNA similar to that of early differentiation, with potential to continue over several weeks. Instead of using preadipocyte cell lines, it would be interesting to determine the dynamic expression of LMNA in adipose tissue- derived stem cells, which are presumed to be pre-programmed to undergo adipogenesis. Such experiments are currently underway.

The size and shape alterations of the nucleus during differentiation are quantifiable and can be used as inputs to develop a model of nuclear morphology over time. A multibody model depicting forces generated on the nucleus was developed. The model took into account the actin filaments reorganizing and dissociating from the nucleus during adipogenic differentiation. This cytoskeletal rearrangement is theorized to reduce tension on the nuclear membrane, allowing the nucleus to reduce in size. Over several simulations, the model demonstrated similar area reduction and accurately predicted the experimental data (see Fig. 7A). Since the mechanical properties of actin filaments are known, the model can also be used to predict the membrane tension. As a result of nucleus becoming smaller, the nuclear components (represented by spherical particles in Fig. 6) must be reorganized to better fit the contracting nucleus. Such nuclear reorganization may induce potential chromosome rearrangement. Chromosome territories have been described as the compartmentalization of DNA in the nucleus, and evidence suggests these high-order arrangements influence gene expression^40,41^. As MSCs reprogram toward a specified lineage, corresponding gene regulation must occur to enhance the physiology of the lineage-specific cell. It is noteworthy to point out Kuroda *et al*. measured the positioning of two chromosomes (12 and 16) during adipogenic differentiation as a non-random event, indicating a directed influence on gene expression^42^. The stiffness of the stem cell nucleus membrane cannot be found in literature with any certainty. However, the static and dynamic balance of forces in the simulation can be used to calculate the membrane stiffness. In the simulation, the initial configuration of the nucleus is generated randomly. The model is given time to settle and achieve a static force balance. Because the stiffness of the actin filaments is known in literature, ${K}_{actin}=0.0437\,N/m$^43^, we can use it to find the nuclear membrane stiffness. Using multiple nucleus configurations, this stiffness was found to be ${K}_{membrane}\approx 0.0055\pm 0.0005\,N/m$, which is approximately an order of magnitude smaller than ${K}_{actin}$. The value calculated for the presented simulation is ${K}_{membrane}=0.0051\,N/m$.

Depolymerization of actin filaments’ effect on the nucleus is hypothesized to function as a reduction of stiffness over time with the formula:

1 $${K}_{Depolymerization}={K}_{Actin}\times {(1-\frac{t}{{t}_{Depolymerization}})}^{n}$$

where *K*_*Depolymerization*_ is the equivalent stiffness of the actin filament during depolymerization process, *t* is time elapsed from the start of the depolymerization process, ${t}_{Depolymerization}=4\,days$ denotes the total depolymerization process time, and *n* determines the rate of depolymerization. Larger values of *n* indicate a more rapid depolymerization at the start of process. It was found that *n* = 4 yielded the best match to the experimental observations, suggesting a very rapid dissociation at the start and slowing down over time.

To further investigate changes in the nuclear shape as a prerequisite for adipogenesis, we utilized microcontact printing of fibronectin to pattern hMSC nuclei into high aspect ratios at the time of cell seeding. This protocol achieved the cell morphology similar to that of microchannels, in which the stretched cell morphology squeezed the nucleus, reducing its roundness (see Fig. 4). As differentiation proceeded on the patterned surfaces, adipogenesis was hindered when compared to that on glass substrate. One possible explanation of the observed results is that the initial overall cell shape and microfilament network is integral to the differentiation potential of MSCs. Alternatively, when the actin polymerizing is stabilized by jasplakinolide, an enhancement of microfilament organization also reduced adipogenesis. Our findings suggest that the mechanical cues modulate the intended differentiation, and the specific cell shapes lend themselves to downstream terminally differentiated cell types (e.g., spindle shape of fibroblast)^44^. The influence of several growth factors on gene regulation has also been described^45,46^, as well as that of substrate mechanical properties^47,48^. This chemical and physical direction of genetic regulation may therefore guide the nuclear shape. In this case, the nuclear shape is a result of – rather than a requirement for – gene regulation, and therefore cannot be forced to encourage differentiation. Forcing the nuclei into a confined shape may prove deleterious to the processes of nuclear remodeling, which should be completed step-wise. The differentiation of MSCs into fully mature adipocytes appear to occur in stages, and stem cells cannot leap previous stages to reach the end more quickly.

In conclusion, we provide evidence for the nuclear reorganization of human MSCs during adipogenic differentiation. We described a significant reduction in the nuclear area along with an increase in the aspect ratio over time. Lamin A/C, the major structural network in the nucleus, is reorganized and upregulated along with this change in shape, suggesting a direct relationship between the LMNA and nuclear morphology. To further elucidate the influence of nuclear shape on differentiation, MSCs with high nuclear aspect ratios were differentiated on microcontact printed patterns. The patterns failed to enhance differentiation, providing evidence for the directed genetic reorganization of stem cells as differentiation continues into distinct, terminally differentiated cell types. Finally, numerical simulations were successfully developed to predict dynamical changes of the nuclei as hMSCs are directed to differentiate to adipocytes.

## Methods

### Cell culture

Human MSCs derived from bone marrow were purchased from Lonza and expanded on tissue culture plates in growth medium (PT-3001, Lonza) until confluence. Once confluent, stem cells were detached from culture plates with 0.5 Trypsin-EDTA and seeded onto glass coverslips at a density of 2.5 × 10^4^ *cells/cm*^2^.

### Adipogenic differentiation

To induce differentiation toward adipocytes, cells were grown in adipogenic differentiation (AD) medium as described previously^10^. AD medium consisted of: High-Glucose Dulbecco’s Modified Eagle Medium (DMEM), 10% fetal bovine serum (FBS), 1% penicillin-streptomycin, 1 *µM* dexamethasone, 200 *µM* indomethacin, 10 *µg/mL* insulin, and 0.5 mM 3-isobutyl-1-methylxanthine (IBMX).

MSCs underwent differentiation for 15 days, with samples being removed for analysis every other day. Samples were fixed in 4 paraformaldehyde for 20 minutes and stained for fluorescence microscopy. Cells were then mounted on to microscope slides and analyzed for nuclei morphology and lipid production.

To examine the influence of actin reorganization on nuclear properties, the actin polymerizing drug jasplakinolide (Santa Cruz Biotech) was used as previously described^49^. MSCs undergoing differentiation were treated with 0.01 *µM* Jasplakinolide in the AD medium, similarly replenished every other day for 15 days.

To confirm any differences in nuclear shape or lipid production are a result of treatment and not cell proliferation dependent, an MTT assay was performed (Sigma). MSCs were seeded on 96-well plates at the density described previously. Cell proliferation of treated cells and control cells were examined following days 1 and 7 of differentiation. Similarly, to prevent cell cycle dependency on differentiating cells of both control and treated groups, cycle synchronization was performed and examined. Cell cycles were synchronized in the G_1_/G_0_ phase using the starvation method as previously described^50^. Briefly, after seeding cells onto coverslips and before differentiation media was added, cells were supplied with growth medium without serum or growth factors for 20 hrs. Following this incubation period, cells were supplied adipogenic medium as specified by treatment group.

### Microcontact-printed surface preparation

To probe whether nuclei shape before differentiation influences the speed and efficiency of maturation, a microcontact-printing technique was performed. Stamping patterns were designed in AutoCAD (Autodesk, San Rafael, CA) and embedded onto a silicon wafer by MuWells (San Diego, CA). Linear patterns were fabricated with a width of 20 *µm* and a depth of 5 *µm*. Polydimethylsiloxane (PDMS) molds were generated with raised design features by mixing 10:1 (w/w) elastomer: crosslinker and pouring onto the silicon wafer. Molds were cured overnight at 60° until hardened. Stamping of fibronectin patterns was performed as described by established protocol^51^. Briefly, PDMS molds were cleaned thoroughly with ethanol, dried with compressed air, and the surfaces containing the stamp features were treated with plasma to promote protein attachment. 50 *µg/mL* of rhodamine-conjugated fibronectin (Cytoskeleton, Denver, CO) was added to the surface of the molds and allowed to bind for 30 minutes. Following binding, the molds were washed with PBS three times and deionized water once. Quickly after washing, the molds were flipped onto 35 mm cell culture dishes and the protein monolayer was allowed to transfer for 5 minutes. After transfer, PDMS molds were carefully peeled off and the dishes were washed with PBS and blocked with 1% F-127 for 30 minutes to prevent cell attachment to the non-stamped surfaces. Excess F-127 was removed with additional washing with PBS and the dishes were incubated overnight in PBS.

### Fluorescence microscopy

Fluorescent microscopy images were taken on a Nikon Eclipse E800 (Melville, NY) at time points of every other day during differentiation. Fluorescent detection included staining for chromatin (NucBlue R37605, Thermofisher) for 15 minutes and lipid deposition (LipidTOX Green H34475, Thermofisher) for 30 minutes. Additionally, immunostaining was performed to observe the nuclear envelope protein, lamin A/C (LMNA). Briefly, cells were fixed in 4 paraformaldehyde for 20 minutes, permeabilized in 0.2% Triton X-100 (Sigma) for 15 minutes and blocked with 3 bovine serum albumin for 30 minutes. Cells were incubated with Alexa-Fluo 594-conjugated anti-LMNA antibodies produced in rabbit (ab215324, Abcam) for 1 h. LMNA antibodies and LipidTOX were diluted in phosphate buffered saline (PBS) at ratios of 1:500 and 1:200, respectively.

### RNA Extraction and reverse transcription-quantitative polymerase chain reaction (RT-qPCR)

Gene profiling after 5 days of adipogenesis in experimental (AD) and control (AC) mediums was performed by reverse transcription-quantitative polymerase chain reaction (RT-qPCR). Briefly, RNA was extracted from cells following the protocol from the Quick RNA mini prep kit (Zymo Research, Irvine, CA). Isolated RNA was transcribed to cDNA using the AzuraQuant cDNA synthesis kit (Azura Genomics, Raynham, MA) according to the manufacturer’s protocol. Quantitative PCR was performed on a 7500 Fast Real-Time PCR System (Applied Biosystems, Foster City, CA) using AzuraQuant Green Fast qPCR Master Mix HiRox. Primers were purchased from Real Time Primers (Elkins Park, PA) and included adipose maturation gene Human peroxisome proliferator-activated receptor gamma (PPARγ) and nuclear membrane protein Human Lamin A/C (LMNA). GAPDH served as the internal reference to normalize the level of gene expression across all samples. Forward and reverse sequence information is recorded in Table 1. Fold changes in gene expression for experimental groups were analyzed relative to untreated controls using the double-delta cycle threshold (2-ΔΔCT) method^52^.

**Table 1:** Forward and reverse primers used for RT-qPCR gene analysis.

Target Gene	Forward Primer	Reverse Primer
PPARγ	5′- GTG CGT GAG GAG TTT AAG GA -3′	5′- GTG CGT GAG GAG TTT AAG GA -3′
LMNA	5′- GTG CGT GAG GAG TTT AAG GA -3′	5′- GTG CGT GAG GAG TTT AAG GA -3′
GAPDH	5′- GAG TCA ACG GAT TTG GTC GT -3′	5′- TTG ATT TTG GAG GGA TCT CG -3′

### Imaging and statistical analysis

Image analysis was done through the fully automated pipeline to compute three major properties: lipid intensity, lamin intensity, and nuclei area. As a preprocessing step all images were normalized by subtraction of the average from all and division by the standard deviation.

Nuclei were segmented by K-means clustering which is an unsupervised learning algorithm popularly used for cell segmentation^23^. There are two classes considered for this step: 1) nuclei, 2) background. After segmentation, the morphology operation is implemented to separate the attached nuclei and removing any remainder of background noise, afterwards detected segments are labeled individually. Labeling the segmented particles gives the number of detected nuclei and makes it possible to extract morphological properties of each particle (nucleus), for this study the area of each nucleus is the focus.

To get the lipid and lamin intensities from the fluorescent images, it’s necessary to remove the background noise. For this purpose, images are filtered by the Gaussian kernel leading to a Gaussian blurred image. A fraction of the Gaussian blurred image is subtracted from the original image as background noise. For background removal there are two parameters that can be modified as needed, the coefficient for Gaussian blurred image and the sigma for the Gaussian filter. All non-zero pixels after background removal are considered as fat or lamin, respectively. To make sure the intensity measure for images from different runs are unified, all the images are normalized by dividing by the maximum pixel value and multiplying with 255, basically they are converted into 8 bit images. Then the intensity per cell is computed by total intensity divided, summation of all pixel values, by the number of cells for each image.

The image analysis pipeline is written in Python 3.5 and open source image analysis libraries OpenCV, Scikit-learn and Scikit-Image has been used. Nuclear morphological characteristics were also determined using ImageJ for comparison purpose. Statistical analysis was performed in Excel (Microsoft, Redmond, WA). Comparison between groups was performed using ANOVA (single factor) with a significance threshold of p < 0.05.

### Dynamic model and simulation

To further elucidate the mechanical forces experienced by the nuclei during adipogenesis, we developed a two-dimensional model of forces acting upon the nucleus as differentiation continues. A model of the nucleus was simulated in MATLAB2018a (MathWorks, Natick, MA).The simulation was generated with the following assumptions: 1) actin filaments keep the nucleus membrane under tension, and 2) this tension dissipates over time due to actin depolymerization and rearrangement, and rearrangement of nucleus’ contents during differentiation. Subsequently, the production of lipids inside the cell drives the later compaction of the nucleus.

The system was modeled as one stem cell nucleus in two dimensions, as shown in Fig. 6. The nucleus membrane is modeled as one continuous spring under tension. All of the nuclear components are modeled as rigid spherical particles. Nucleoplasm is modeled as a liquid exerting drag forces on the rigid particles, effectively damping the particles’ movement. The cytoskeletal forces are represented by actin filaments attached to the nuclear membrane. The actin filaments are modeled as springs keeping the membrane under tension. The number of attached actins (i.e., 16) is modeled to be equal to the number of beads in the periphery of nucleus as shown in Fig. 6B. The effects of rearrangement and depolymerization of the cytoskeletal structure are modeled as the reduction in actin filaments stiffness over time. The rate is governed by the Eq. 1. The actin stiffness reaches the value of zero after the 5^th^ day. The nucleus is disconnected from the cytoskeleton at this point. The effect of lipid accumulation is modeled as external forces acting on the nucleus.

Given the known values of actin filament stiffness and nucleus area we can estimate the stiffness of the nucleus membrane. This is based on the static and dynamic balance of forces between the membrane and actin filaments before differentiation. Additionally, observing the change in membrane area versus nuclear membrane tension, we can determine the rate of actin rearrangement and depolymerization. Finally we can estimate the amount of force lipid accumulation exerts on the nucleus based on the change in the nucleus area in the later stages.

The dynamic model consists of 250 rigid spherical particles inside the nucleus representing the chromosomes and proteins. Using the nucleus density and allowing for the mass of the nucleoplasm, the mass of each particle is calculated to be $m=21.8672\times {10}^{-18}\,kg=21.8672\,fg$ with a radius of $\,r=123.1\,nm$. A total of six forces act on the particles. Contact, viscous damping, and the random forces associated with Brownian motion act on all particles, while the actin, membrane and external forces act only on the particles in the periphery of the nucleus. Applying Newton’s second law to a representative particle yields,

2 $$m\,\ddot{{\boldsymbol{x}}}+\beta \,\dot{{\boldsymbol{x}}}=\sum {\boldsymbol{F}}$$

where ${\boldsymbol{x}}$ contains the generalized coordinates, and $\ddot{{\boldsymbol{x}}}$ and $\dot{{\boldsymbol{x}}}$ are generalized acceleration and velocity. The mass of one particle is *m*. The term $\beta =1.39\times {10}^{-8}kg/s$ is the coefficient of viscous friction for nucleoplasm. The sum of all other forces is $\,\sum {\boldsymbol{F}}$ which includes contact forces, random forces associated with Brownian motion, membrane tension forces, Actin forces, and external forces resulting from lipid accumulation.

The simulation of nucleus contraction using Eq. 2 in molecular dynamics simulations is prohibitively expensive computationally. The forces involved are many orders of magnitude larger than the masses. That will cause large accelerations that drastically increase computational time. This is a common issue with all molecular dynamics simulations. For Eq. 2, it will take one minute of CPU time to model one nanosecond of real time. Because the contraction of the nucleus was observed over 15 days, it is infeasible to obtain a simulation by simply applying standard numerical integration techniques to Eq. 2, as in molecular dynamics simulation.

In this work, a scaling approach is used, which is based on the method of multiple scales (MMS)^53^. The premise is that large accelerations produce high frequency vibrations, on the order of 1/picoseconds, which have diminishing effects on the long term system behavior, on the order of days and weeks. Thus, the model can be scaled to remove the high frequency component of the motion when it is desired to observe the system behavior over longer time periods. The MMS uses an asymptotic expansion to decompose Eq. 1 into different time scales, *T*_*i*_, based on ${T}_{i}={e}^{i}\,t$, where *e* is a small number obtained from the model characteristics. Examination of the *e*^0^ term, the zero order perturbation^54^, suggests a cancellation of the generalized active forces in the model. These cancelled forces can be removed from the dynamic model by scaling the generalized active forces. In this case, two scaling factors are needed because of the imbalances between viscous damping and the remaining forces. These dimensionless scaling factors are ${a}_{2}\approx O(\beta /{K}_{actin})$ and ${b}_{2}\approx O(m/\beta )$.

The resulting scaled dynamic model has the form:

3 $$m\ddot{{\boldsymbol{x}}}+{a}_{2}\,\beta \,\dot{{\boldsymbol{x}}}\,=({a}_{2}{b}_{2})\,\sum {\boldsymbol{F}}$$

where the dimensionless scaling factors are ${a}_{2}=3.2\times {10}^{-10}$, and ${b}_{2}=1.6\times {10}^{-12}$. The unit system is, time in 10^3^ *s* = 1 *ks*, mass in ${10}^{-18}\,kg=1\,fg$, and length in $1\,\mu m$. All of the terms in Eq. 3 are in proportion in the selected unit system. Therefore, the system can be simulated in exceptionally fast time. Total CPU time is $4738.3\,s\approx 1.5\,hours$ for 15 days of observation. This represents a computation time reduction on the order of 10^14^; the computational time for a standard numerical integration would be beyond hundreds of years. This drastic reduction in computational time allows an exploration of the physical properties of the nucleus such as the membrane stiffness and the actin depolymerization rate described in Eq. 1. The value of the parameters used in the simulation are included in Table 2. Details of the computer simulation are more thoroughly discussed elsewhere^55^.

**Table 2:** Additional Parameters in Computer Simulation.

Parameter	Definition	Value	Change
ρ	Nuclear density	$1400\,kg.{m}^{-3}$ ^56^	constant
η	nucleoplasm viscosity	$6\times {10}^{-3}\,kg.{m}^{-1}.{s}^{-1}$ ^57^	constant
kB	Boltzmann constant	$1.380\times {10}^{-23}J.{K}^{-1}$	constant
T	temperature	$310.15\,K$	constant
Kactin	actin stiffness	$0.0437\,kg.{s}^{-2}$ ^43^	Changes with the Eq. 1
